# Assessment of the Optic Disc Morphology Using Spectral-Domain Optical Coherence Tomography and Scanning Laser Ophthalmoscopy

**DOI:** 10.1155/2014/275654

**Published:** 2014-07-06

**Authors:** Pilar Calvo, Antonio Ferreras, Beatriz Abadia, Mirian Ara, Michele Figus, Luis E. Pablo, Paolo Frezzotti

**Affiliations:** ^1^Ophthalmology Department, Miguel Servet University Hospital, Aragon Health Sciences Institute, Isabel la Catolica 1–3, 50009 Zaragoza, Spain; ^2^Department of Surgery, Ginecology and Obstetrics, University of Zaragoza, 50009 Zaragoza, Spain; ^3^Department of Neurosciences, University of Pisa, 56126 Pisa, Italy; ^4^Department of Ophthalmology, University of Siena, 53100 Siena, Italy

## Abstract

*Objective*. To compare the equivalent optic nerve head (OHN) parameters obtained with confocal scanning laser ophthalmoscopy (HRT3) and spectral-domain optical coherence tomography (OCT) in healthy and glaucoma patients. *Methods*. One hundred and eighty-two consecutive healthy subjects and 156 patients with open-angle glaucoma were divided into 2 groups according to intraocular pressure and visual field outcomes. All participants underwent imaging of the ONH with the HRT3 and the Cirrus OCT. The ONH parameters and the receiver operating characteristic (ROC) curves were compared between both groups. *Results*. Mean age did not differ between the normal and glaucoma groups (59.55 ± 9.7 years and 61.05 ± 9.4 years, resp.; *P* = 0.15). Rim area, average cup-to-disc (C/D) ratio, vertical C/D ratio, and cup volume were different between both instruments (*P* < 0.001). All equivalent ONH parameters, except disc area, were different between both groups (*P* < 0.001). The best areas under the ROC curve were observed for vertical C/D ratio (0.980 for OCT and 0.942 for HRT3; *P* = 0.11). Sensitivities at 95% fixed-specificities of OCT parameters were higher than those of HRT3. *Conclusions*. Equivalent ONH parameters of Cirrus OCT and HRT3 are different and cannot be used interchangeably. ONH parameters measured with OCT yielded a slightly better diagnostic performance.

## 1. Introduction

Identification of damage to the optic nerve head (ONH) is key for glaucoma diagnosis. For more than 10 years, the confocal scanning laser ophthalmoscope Heidelberg Retina Tomograph (HRT, Heidelberg Engineering, Heidelberg, Germany) has been widely used to evaluate the optic disc morphology and monitor changes over time [1–10].

On the other hand, since the introduction of optical coherence tomography (OCT) in 1993 [11, 12], this technique has been rapidly adopted into clinical practice and is now one of the main diagnostic methods in ophthalmology. The OCT is a computer-assisted precision instrument that delineates cross-sectional anatomy of the retina and provides in vivo real time images of different structures of the eye. The performance of OCT has been constantly improved since its introduction, and the latest versions can generate three-dimensional images from multiple* A*-scans acquired on the optic disc. The cube of data generated from these* A*-scans enables a far more extensive evaluation of the peripapillary area including retinal nerve fiber layer profiles, en face images (fundus image), and ONH assessment. Diagnostic ability of OCT for glaucoma diagnosis has been reported in the past [13–22]. Nevertheless, few studies have compared the ONH measurements between these devices [23, 24].

The purpose of this study was to compare the equivalent ONH parameters obtained with Cirrus OCT (Carl Zeiss Meditec, Dublin, CA) and HRT3 in healthy individuals and glaucoma patients as well as evaluate their accuracy for glaucoma diagnosis.

## 2. Materials and Methods

The study protocol adhered to the tenets of the Declaration of Helsinki and was approved by the Clinical Research Ethics Committee of Aragón (CEICA).

A total of 350 eyes of 350 subjects were prospectively preenrolled. Normal eyes were consecutively recruited from patients referred for refraction that underwent routine examination without abnormal ocular findings, hospital staff, and relatives of patients in our hospital. The glaucoma group comprised subjects with primary open-angle glaucoma, pseudoexfoliative glaucoma, and pigmentary glaucoma. Patients with glaucoma were recruited consecutively from an ongoing longitudinal follow-up study at the Miguel Servet University Hospital. In 4 cases we could not obtain a reliable standard automated perimetry (SAP) after 3 attempts and in 8 cases the subject did not complete the visits included in the study protocol. These 12 subjects were excluded from further analysis. Finally, 338 eyes of Caucasian origin were included in the statistical analysis. When both eyes fulfilled the inclusion criteria, only one eye per subject was randomly selected.

All of them had to meet the following inclusion criteria: best-corrected visual acuity (BCVA) better than 20/30 (Snellen), refractive error less than 5 spherical diopters and 2 diopters of cylinder, transparent ocular media (nuclear colour/opalescence, cortical or posterior subcapsular lens opacity < 1) according to the Lens Opacities Classification System III system [25], and open-anterior chamber angle. Subjects with previous intraocular surgery, diabetes or other systemic diseases, history of ocular or neurologic disease, or current use of a medication that could affect visual field sensitivity were excluded.

Participants underwent full ophthalmologic examination: clinical history, best-corrected visual acuity, biomicroscopy of anterior segment using a slit lamp, gonioscopy, Goldmann applanation tonometry, central corneal ultrasonic pachymetry (OcuScan RxP; Alcon Laboratories Inc., Irvine, Ca), and ophthalmoscopy of the posterior segment.

At least 2 reliable SAPs were performed to minimize the learning effect [26–28]. The visual field was evaluated with a Humphrey Field Analyzer, model 750i (Zeiss Humphrey Systems, Dublin, CA), by using the 24-2 SITA Standard strategy. Near addition was added to the subject's refractive correction. If fixation losses were higher than 20% or false-positive or false-negative rates were higher than 15%, the test was repeated. The subjects completed the perimetry measurements prior to any structural test, and each perimetry test was performed at least 3 days apart to avoid a fatigue effect. Abnormal SAP results were defined as typical glaucomatous defects with a pattern standard deviation significantly elevated beyond the 5% level and/or a Glaucoma Hemifield Test outside normal limits.

The sample was divided into 2 groups according to the intraocular pressure (IOP) and visual field outcome, regardless of optic disc appearance. Glaucomatous eyes had an IOP of greater than 21 mmHg and abnormal SAP results.

Topographic analysis of the optic disc was performed using the HRT3, which provides topographic measurements of the ONH derived from 16 to 64 optical sections to a depth of 4 mm. The spherical equivalent refractive error of each eye was adjusted in the dioptric ring of the HRT3. Magnification errors were corrected by the software based on keratometric readings. Topographic images were then obtained through dilated pupils (1% tropicamide eye drops; Alcon Laboratories Inc., Fort Worth, TX) and analysed with the Advanced Glaucoma Analysis 3.1 software. All scans had to have an interscan standard deviation of less than 30 *μ*m. The margin of the optic disc was manually traced by the same glaucoma specialist, who was masked to the patients' identity and clinical history, defining the inner edge of Elschnig's ring with at least a four-point contour line. All scans had to have an interscan standard deviation of less than 30 *μ*m. The global stereometric parameters investigated in this study were rim area, disc area, average cup/disc (C/D) ratio, vertical C/D ratio, and cup volume.

The equivalent ONH parameters were also measured using the Cirrus OCT (Optic Disc Cube 200 × 200 scan protocol; software version 6.2) following a standard procedure described elsewhere. Left eye data were converted to a right eye format. All images were acquired with a quality greater than 6/10. The same operator performed all scans with the same device.

All the ophthalmic examinations, perimetry tests, and the topographic analysis of the ONH were performed within 6 weeks of the subject's date of enrolment into the study.

The statistical analyses were calculated using MedCalc (version 12; MedCalc software, Mariakerke, Belgium) and IBM SPSS statistical software (version 22; IBM Corporation, Somers, NY). All the variables followed a normal distribution as verified with the Kolmogorov-Smirnov test (K-S of 1 sample). Demographics, HRT3, and OCT parameters were compared between both groups with the independent *t*-test.

The receiver operating characteristic (ROC) curves were plotted for the ONH parameters acquired with both devices. The best areas under the ROC curves (AUCs) were also compared (DeLong method) [29].

## 3. Results

Table 1 shows the clinical characteristics of the study sample. Mean age in the normal group (182 eyes) was 59.55 ± 9.71 years, while mean age in the glaucoma group (156 eyes) was 61.05 ± 9.43 years (*P* = 0.15). There were significant differences (*P* < 0.001) in mean IOP, best-corrected visual acuity, central corneal thickness, mean deviation of SAP, pattern standard deviation of SAP, and the visual field index (VFI) of SAP between both groups.

Disc area measured with Cirrus OCT did not differ between the groups (*P* = 0.10). Nevertheless, HRT3 showed significant differences for disc area between the normal and glaucoma groups (*P* < 0.001). Rim area, average C/D ratio, vertical C/D ratio, and cup volume of both Cirrus OCT and HRT3 were different between both groups (*P* < 0.001).

Table 2 shows the equivalent ONH parameters measured with Cirrus OCT and HRT3 in the normal and glaucoma groups. In the normal group, average and vertical C/D ratios were different between Cirrus OCT and HRT3, while in the glaucoma group, all the equivalent ONH parameters were different (*P* < 0.001).

The largest AUCs obtained with Cirrus OCT (Table 3) were observed for vertical C/D ratio (0.980; *P* < 0.001) and rim area (0.966; *P* < 0.001). All AUCs of Cirrus OCT were above 0.92, except disc area. For HRT3, the largest AUCs were observed for vertical C/D ratio (0.942; *P* < 0.001) and rim area (0.905; *P* < 0.001). The differences of the AUCs between both devices were tested by the DeLong method (Table 4). No differences were found for the best ONH parameters (vertical C/D ratio) to discriminate between healthy and glaucoma patients (*P* = 0.11).

Sensitivities of all ONH parameters ranged from 79% to 98% at 86% to 99% specificities (Table 5). The best sensitivity-specificity balance was observed for vertical C/D ratio in both devices: 94%-93% for Cirrus OCT and 90%–94% for HRT3. The rim area (Cirrus OCT) and vertical C/D ratio (HRT3) yielded the highest sensitivities (96.1%) at 85% fixed-specificity, whereas the sensitivities were 80.7% and 91%, respectively, at 95% fixed-specificity. Overall, sensitivities at 95% fixed-specificities of the Cirrus OCT parameters were higher than those of HRT3.

## 4. Discussion

This study was aimed at comparing the equivalent ONH parameters obtained by Cirrus OCT and HRT3 (rim area, disc area, average C/D ratio, vertical C/D ratio, and cup volume) between healthy and glaucoma patients. We found significant differences for average C/D ratio, vertical C/D ratio, and cup volume in the normal group and for all equivalent ONH parameters in the glaucoma group between both instruments.

Few articles have compared the ONH parameters between Cirrus OCT and HRT3. Sato et al. [23] performed a similar study by comparing ONH parameters from 96 glaucoma patients and 21 healthy subjects. They also found significant differences (*P* < 0.001) in all the studied parameters, although this analysis was performed on the total sample, not for subgroups.

The software of the HRT3 determines a reference plane parallel to the retina surface. The standard reference plane is located 50 *μ*m posterior to the temporal ONH margin. The tissue above the reference plane is considered as “rim” while the structure below is considered as “cup.” Although the level of the reference plane should not affect the measurement of the disc area, its position has a direct impact on the calculation of the rim and cup areas. OCT measurement does not depend on reference planes and does not require prior manual outlining of disc boundaries, reducing the dependency on operator skill. Additionally, in Cirrus OCT, the disc edge is determined by the termination of Bruch's membrane; thus, the measurement of the rim and cup areas corresponds with the actual anatomy in the same plane as the optic disc. HRT3 does not take into account the inclination of the optical disc as it acquires the images in horizontal planes, leading to a worse performance in tilted discs compared to OCT.

Other authors have evaluated the differences for ONH parameters obtained by Stratus OCT and HRT [30–32]. Generally, it is accepted that Stratus OCT tended to measure larger areas of the disc than HRT. In our work we did not observe the same trend: disc area was 1.93 mm^2^ for Cirrus OCT and 1.87 mm^2^ for HRT3 (*P* = 0.17) in healthy individuals. In the glaucoma group, disc area was 2.02 mm^2^ for Cirrus OCT and 2.11 mm^2^ for HRT3 (*P* = 0.01). By contrast, in the work of Sato et al. [23] disc areas with Cirrus OCT were smaller than those obtained with the HRT (1.97 mm^2^ versus 2.27 mm^2^). They included a small sample in the normal group (*n* = 21) and performed the statistical analysis in the whole sample, including normal and glaucomatous eyes. Larger samples usually lead to more precise estimates.

Moghimi et al. [24] also studied the relationship between the ONH parameters measured with Cirrus OCT and HRT3. This work included 13 healthy individuals, 21 glaucoma suspects, and 37 patients with established glaucoma. They reported that HRT3 tended to overestimate disc and rim areas compared to Cirrus OCT.

Regarding the ONH parameters we found statistical differences for all the ONH parameters, except disc area, between the normal and glaucoma groups for both devices. These findings are consistent with previous studies [33–35]. Mwanza et al. [35] also found differences for all ONH parameters, except for disc area. They included 73 patients with glaucoma (31 mild, 14 moderate, and 28 severe glaucomas) and 146 healthy patients. They found AUC values ranging from 0.901 to 0.963 for all parameters, except disc area. They also observed, in agreement with our findings, that vertical C/D ratio was one of the best parameters to discriminate between healthy and glaucomatous eyes.

The best HRT3 parameter to differentiate between healthy and glaucoma patients was also vertical C/D ratio (0.942). de León-Ortega et al. [36] found a similar result, although their AUC value was lower (0.861). Obviously, the severity of visual field loss has an important influence on imaging instrument sensitivity [27, 37]. More severe disease is associated with increased sensitivity. In our study, most glaucomatous eyes had mild to moderate visual field defects, according to the Hodapp-Parrish-Anderson score [38].

The quality of the data obtained by the imaging devices is influenced by the media opacity, retinal pigment epithelium status, instrument variability, and positioning and centering of the images. Our sample had transparent ocular media, but this condition is not always possible in clinical practice. Clinicians should take into account that the accuracy of the measurements is related to the quality of the images.

## 5. Conclusions

ONH parameters of Cirrus OCT and HRT3 should not be used interchangeably. Optic disc assessment using Cirrus OCT showed a slightly better diagnostic performance than HRT3 to discriminate between healthy and glaucoma patients.

## Figures and Tables

**Table 1:** Clinical characteristics of the sample.

	Normal		Glaucoma		*P*
	Mean	SD	Mean	SD	
Age (yrs)	59.55	9.7	61.05	9.4	0.152∗
BCVA (Snellen)	0.93	0.1	0.82	0.2	<0.001∗
IOP (mmHg)	17.10	2.3	26.68	5.6	<0.001∗
Pachymetry (*μ*m)	554.63	33.7	535.30	37.7	0.001∗
MD SAP (dB)	−0.38	0.9	−6.64	6.0	<0.001∗
PSD SAP	1.42	0.3	6.03	3.8	<0.001∗
VFI SAP	99.55	0.7	83.97	17.2	<0.001∗
Gender (male/female)	57/125		68/88		0.031∗∗
*N*	182		156		

*Student's *t*-test; **chi-square test; BCVA: best-corrected visual acuity; IOP: intraocular pressure; MD: mean deviation; PSD: pattern standard deviation; SAP: standard automated perimetry; VFI: visual field index; SD: standard deviation; *N*: number of cases.

**Table 2:** Comparison of the equivalent ONH parameters measured with Cirrus OCT and HRT3 in the normal and glaucoma groups.

		Cirrus OCT				HRT3				P*
		Min	Max	Mean	SD	Min	Max	Mean	SD	
Normal group	Rim area	0.87	2.33	1.49	0.29	0.86	2.59	1.50	0.32	0.87
	Disc area	1.07	2.67	1.93	0.32	0.89	3.43	1.87	0.44	0.17
	Average C/D	0.06	0.77	0.42	0.17	0.00	0.58	0.19	0.13	<0.001
	Vertical C/D	0.06	0.74	0.40	0.16	0.00	0.74	0.26	0.22	<0.001
	Cup volume	0.00	0.78	0.10	0.13	0.00	0.48	0.07	0.09	0.050

Glaucoma group	Rim area	0.23	1.45	0.80	0.25	0.20	2.09	1.06	0.34	<0.001
	Disc area	1.45	2.53	2.02	0.26	1.30	3.82	2.11	0.41	0.013
	Average C/D	0.53	0.9	0.76	0.09	0.00	0.87	0.49	0.18	<0.001
	Vertical C/D	0.54	0.89	0.74	0.09	0.00	0.95	0.66	0.21	<0.001
	Cup volume	0.02	0.97	0.49	0.23	0.00	1.41	0.31	0.22	<0.001

*Student's *t*-test.

C/D: cup-to-disc ratio; Min: minimum; Max: maximum; SD: standard deviation.

**Table 3:** Areas under the ROC curve of the optic nerve head parameters to discriminate between healthy and glaucoma patients.

		AUC	SE	*P*	CI 95%	
					Inferior limit	Superior limit
Cirrus OCT	Rim area	0.966	0.02	<0.001	0.936	0.995
	Disc area	0.584	0.05	0.120	0.481	0.688
	Average C/D	0.961	0.02	<0.001	0.932	0.991
	Vertical C/D	0.980	0.01	<0.001	0.960	0.999
	Cup volume	0.924	0.03	<0.001	0.873	0.975

HRT3	Rim area	0.905	0.03	<0.001	0.849	0.962
	Disc area	0.642	0.05	0.009	0.544	0.740
	Average C/D	0.887	0.03	<0.001	0.824	0.950
	Vertical C/D	0.942	0.03	<0.001	0.888	0.995
	Cup volume	0.902	0.03	<0.001	0.846	0.958

C/D: cup-to-disc ratio; AUC: area under the receiver operating characteristic curve; CI: confidence interval; SD: standard error.

**Table 4:** Comparison of the best areas under the ROC curve of the equivalent optic nerve head parameters between Cirrus OCT and HRT3 (DeLong method).

		Cirrus OCT		
		Rim area	Vertical C/D ratio	Cup volume
HRT3	Rim area	0.015	0.003	0.318
	Vertical C/D ratio	0.363	0.114	0.489
	Cup volume	0.017	0.001	0.233

C/D: cup-to-disc.

**Table 5:** Best sensitivity-specificity balance of Cirrus OCT and HRT3 to discriminate between normal and glaucomatous eyes.

	Optimal cut-off point	Sensitivity (%)	Sensitivity 95% CI	Specificity (%)	Specificity 95% CI	Sensitivity at fixed specificity	
						Specificity 85%	Specificity 95%
Cirrus OCT							
Rim area	≤1.2	98.1	90.1–100	86.3	76.2–96.2	96.1	80.7
Average C/D	>0.67	83.3	70.7–92.1	98.9	92.6–100	85.2	84.6
Vertical C/D	>0.59	94.2	84.6–98.8	93.4	84.7–97.7	77.5	86.5
Cup volume	>0.263	87.1	75.1–94.6	89.0	79.5–95.1	91	72.4
HRT3							
Rim area	≤1.16	79.4	66.5–89.4	90.1	81.2–96.1	83.3	68.5
Average C/D	>0.39	86.5	74.7–94.5	94.5	86.6–98.5	90.3	80.7
Vertical C/D	>0.55	90.3	79.7–96.9	94.5	86.6–98.5	96.1	91
Cup volume	>0.14	83.3	70.7–92.1	86.3	76.2–93.2	81.4	59.6

C/D: cup-to-disc ratio; CI: confidence interval.
